# Firmicutes/Bacteroidetes Ratio as an Insufficient Indicator of Metabolic Status in Mexican Young Adults

**DOI:** 10.3390/nu18071084

**Published:** 2026-03-28

**Authors:** Ana Teresa Nez-Castro, Luis Guillermo González-Olivares, Laura Berenice Olvera-Rosales, Edwin Gualberto Barrón-Calva, Edwin Alonso Chávez-Mejía, Arianna Omaña-Covarrubias, Carlos Manuel Franco-Abuín, Alicia del Carmen Mondragón-Portocarrero

**Affiliations:** 1 Instituto de Ciencias de la Salud, Área Académica de Nutrición, Universidad Autónoma del Estado de Hidalgo, Circuito Ex Hacienda, La Concepción S/N, Carretera Pachuca Actopan, San Agustín Tlaxiaca 42060, Hidalgo, Mexico; teresa_nez@uaeh.edu.mx; 2 Instituto de Ciencias Básicas e Ingeniería, Área Académica de Química, Universidad Autónoma del Estado de Hidalgo, Carr. Pachuca-Tulancingo km. 4.5, Pachuca 42184, Hidalgo, Mexico; lgonzales@uaeh.edu.mx (L.G.G.-O.); ol232998@uaeh.edu.mx (L.B.O.-R.); 3 Instituto de Ciencias Sociales y Humanidades, Área Académica de Sociología y Demografía, Universidad Autónoma del Estado de Hidalgo, Carr. Pachuca-Actopan km. 4.5 S/N, Pachuca 42084, Hidalgo, Mexico; edwin_barron@uaeh.edu.mx; 4 Instituto de Ciencias de la Salud, Área Académica de Medicina, Universidad Autónoma del Estado de Hidalgo, Circuito Ex Hacienda, La Concepción S/N, Carretera Pachuca Actopan, San Agustín Tlaxiaca 42060, Hidalgo, Mexico; edalo@outlook.es; 5 Laboratorio de Higiene, Inspección y Control de Alimentos, Departamento de Química Analítica, Nutrición y Bromatología, Campus Terra, Universidade da Santiago de Compostela, 27002 Lugo, Spain; carlos.franco@usc.es

**Keywords:** Firmicutes/Bacteroidetes ratio, intestinal microbiota, body fat percentage, metabolic indicators

## Abstract

**Background:** The Firmicutes/Bacteroidetes (F/B) ratio has been proposed as a microbial biomarker of obesity and metabolic alterations; however, its reliability remains controversial, particularly in young populations. **Methods:** This study evaluated the relationship between the F/B ratio, body fat percentage, and metabolic markers in 70 university students aged 18–25 years, classified as normal weight (29.5%), overweight (27.4%), or obese (43.2%). Anthropometric measurements and biochemical parameters (glucose, triglycerides, total cholesterol, and HDL-C) were obtained using standard methods, and stool samples were analyzed to determine the F/B ratio. **Results:** Mean glucose and cholesterol were within normal ranges, whereas triglycerides showed high variability, and HDL-C was lower in men. Although the F/B ratio increased across nutritional groups, regression analyses showed weak correlations (R < 0.5) and no significant associations (*p* > 0.05). **Conclusions:** The F/B ratio is not an adequate standalone indicator of metabolic status in Mexican young adults.

## 1. Introduction

Excessive accumulation of body fat remains a major global public health concern. In Mexico, the 2018 National Health and Nutrition Survey reported that 75.2% of adults are affected by overweight or obesity, a prevalence that continues to rise and significantly increases the risk of chronic conditions such as hypertension, diabetes, cancer, and musculoskeletal disorders [1]. According to the World Health Organization, these disorders are largely driven by an imbalance between caloric intake and energy expenditure, resulting in adiposity and metabolic dysregulation [2]. In recent years, growing attention has focused on the gut microbiota as a potential contributor to these processes through its roles in energy metabolism, adipose tissue regulation, and appetite-related hormonal signaling [3].

Several studies have reported that obesity is frequently associated with alterations in gut microbiota composition, commonly referred to as dysbiosis, characterized by shifts in bacterial diversity and relative abundance [4,5]. One of the most widely described patterns of dysbiosis is a reduction in Bacteroidetes accompanied by an increase in Firmicutes, a profile initially proposed as a hallmark of obesity [6]. Supporting this concept, Koliada et al. observed a positive association between body mass index (BMI) and the Firmicutes/Bacteroidetes (F/B) ratio in a Ukrainian population, suggesting its potential use as a biomarker of metabolic risk [7].

Firmicutes comprises a large, diverse phylum with more than 200 genera, many of which have an enhanced capacity to ferment complex carbohydrates and increase dietary energy harvest, potentially contributing to weight gain [8,9]. Conversely, the Bacteroidetes phylum—primarily represented by *Bacteroides*, *Prevotella*, and *Porphyromonas* genera—plays important roles in immune modulation, short-chain fatty acid production, and the regulation of metabolic pathways involved in energy balance [10,11,12]. Reduced abundance of Bacteroidetes has been associated with increased adiposity and adverse metabolic profiles.

However, although these associations have been widely reported, attributing specific metabolic functions at the phylum level may be overly simplistic. Functional capabilities within both Firmicutes and Bacteroidetes are highly heterogeneous and are more accurately interpreted at the genus or species level. Therefore, while the Firmicutes/Bacteroidetes (F/B) ratio has been proposed as a biomarker of dysbiosis, its biological interpretation remains limited and strongly context-dependent [10,12].

Despite these observations, the relationship among gut microbiota composition, adiposity, and metabolic markers remains complex and highly context-dependent. Factors such as diet, lifestyle, and host genetics strongly shape microbiota structure and function [13,14], potentially modulating its effects on host energy regulation and metabolic health [15]. Kim et al. reported associations between microbiota alterations and abnormal serum glucose, triglyceride, and cholesterol levels in overweight and obese individuals, further supporting a link between gut microbiota and metabolic regulation [16]. However, these associations are not consistently observed across populations, age groups, or metabolic phenotypes.

Beyond adiposity, the F/B ratio has been proposed to influence metabolic pathways involved in inflammation, insulin resistance, and lipid metabolism. Lipopolysaccharides (LPSs) are structural parts of Gram-negative bacteria, mainly linked to members of the Bacteroidetes phylum rather than Firmicutes. Therefore, connections between gut microbiota composition and inflammatory processes, such as the low-grade chronic inflammation seen in obesity, should not be directly inferred from the Firmicutes/Bacteroidetes (F/B) ratio alone, as this metric does not reflect functional or taxonomic specificities related to endotoxin production [5,13]. Additionally, dietary patterns rich in fats and refined carbohydrates may further elevate the F/B ratio, exacerbating metabolic disturbances and reinforcing interactions among diet, microbiota composition, and host metabolism [4,14]. Nonetheless, emerging evidence suggests that the F/B ratio alone may oversimplify the complexity of the gut microbiota and may not adequately capture early metabolic alterations, particularly in young adults.

Therefore, this study aimed to compare the Firmicutes/Bacteroidetes (F/B) ratio with body fat percentage and metabolic indicators among young university students categorized as normal weight, overweight, or obese.

## 2. Materials and Methods

### 2.1. Participants

The sample size was initially estimated based on population proportion criteria from previous studies. However, considering the comparative nature of this study, this approach might not fully capture the statistical power needed for between-group analyses. The sample calculation for the groups to be evaluated was performed using the following formula:

$$n=\frac{N\times \;{Z\alpha }^{2}\times p\times q}{d^{2}\times \left(N-1\right)+\;Z\alpha^{2}*p*q}$$

$$n=\frac{134\times 0.45\times 0.55}{0.03^{2}\times \left(134-1\right)+1.96^{2}\times 0.45\times 0.55}$$

where

*N* = total population

*Z*α^2^ = 1.962 (95% security)

*p* = expected proportion (45% = 0.45)

*q* = 1 − *p* (0.55)

*d* = precision (3%)

Based on the calculated sample size, a stratified random sample of 130 university students was initially chosen from the Institute of Health Sciences at the Autonomous University of the State of Hidalgo. Participants had to be 18–25 years old, classified as having normal weight, overweight, or obesity based on body fat percentage, and must not have used antibiotics in the two weeks before sampling. Exclusion criteria included menstruation, pregnancy, or lactation in women, as well as medical conditions affecting adiposity, such as dyslipidemia, insulin resistance, or type 1 or type 2 diabetes. After applying these criteria, 70 participants were included in the final analysis.

### 2.2. Study Design and Anthropometric Data Collection

This observational, cross-sectional, and comparative study examined the relationship between body fat percentage, glucose, triglycerides, total cholesterol, HDL cholesterol, and the Firmicutes/Bacteroidetes (F/B) ratio across three groups of university students categorized as normal weight, overweight, or obese. Participants were classified by body mass index (BMI) according to World Health Organization criteria. Additionally, classification by body fat percentage (%FM) was performed using sex-specific reference ranges from the InBody 270 bioimpedance device (InBody Co., Ltd., Seoul, Republic of Korea), enabling a more direct assessment of adiposity. The study aimed to identify potential links between these metabolic and microbiota-related variables. Anthropometric measurements were taken using standardized procedures. Height was measured with a portable stadiometer (Seca^®^ 213, Seca GmbH & Co. KG, Hamburg, Germany) in accordance with ISAK guidelines. %FM was measured using a bioelectrical impedance analyzer (InBody 270), following the manufacturer’s standardized protocol. This method estimates body composition based on electrical conductivity and has been widely used in clinical and research settings, although it may be influenced by hydration status and physiological variability.

### 2.3. Biochemical Parameters

Participants fasted for 8 h before sample collection. Venous blood was drawn, labeled, refrigerated, and centrifuged for 10 min at 3400 rpm using a VanGuard V 6500 centrifuge (Hamilton Bell^®^, Montvale, NJ, USA). The resulting serum was transported on ice to the laboratory for subsequent biochemical analyses.

#### 2.3.1. Glucose

Fasting glucose levels were measured using the hexokinase enzymatic method, a reference procedure for clinical plasma glucose quantification. In this assay, hexokinase catalyzes the phosphorylation of glucose to glucose-6-phosphate, which is then oxidized by glucose-6-phosphate dehydrogenase, reducing nicotinamide adenine dinucleotide phosphate oxidized (NADP^+^) to Nicotinamide adenine dinucleotide phosphate (NADPH). The photometric measurement of NADPH at 340 nm was used to determine glucose concentration, providing high specificity and accuracy. 

#### 2.3.2. Triglycerides

Triglycerides were measured using the enzymatic glycerol phosphate oxidase–peroxidase (GPO-POD) method. Lipoprotein lipase hydrolyzes triglycerides into glycerol and free fatty acids; glycerol is then phosphorylated by glycerol kinase, and the resulting glycerol-3-phosphate is oxidized to produce hydrogen peroxide. A peroxidase-catalyzed chromogenic reaction creates a color proportional to triglyceride levels. 

#### 2.3.3. Total Cholesterol

Total cholesterol was measured using the enzymatic cholesterol oxidase/peroxidase (CHO-POD) method. Cholesterol esterase first hydrolyzes esterified cholesterol; then, free cholesterol is oxidized by cholesterol oxidase to produce cholest-4-en-3-one and hydrogen peroxide. Hydrogen peroxide reacts with chromogenic substrates under peroxidase catalysis to form a colored compound that correlates with cholesterol levels.

#### 2.3.4. HDL Cholesterol

HDL-C was measured using a homogeneous detergent-based enzymatic assay. Non-HDL-C lipoproteins were selectively masked or inactivated with agents such as PEG or cyclodextrins, allowing only HDL-C particles to participate in enzymatic reactions. Cholesterol esterase and cholesterol oxidase act on HDL-C, and the produced hydrogen peroxide engages in a peroxidase-mediated chromogenic reaction. Absorbance directly indicates HDL-C concentration, following the manufacturer’s instructions.

### 2.4. Fecal Samples and DNA Extraction

Participants provided their first fecal sample of the day in a sterile, screw-cap container. Immediately after collection, samples were stored at −18 °C to preserve microbial integrity until laboratory processing. For microbial DNA extraction, frozen samples were thawed and processed using the Bio Basic Genomic DNA Extraction Kit (Thermofisher Scientific, Willmington, DE, USA) following the manufacturer’s protocol. The extracted DNA was aliquoted into sterile Eppendorf tubes and stored at −18 °C until further analysis.

Before PCR amplification, DNA quality and purity were checked using a Nanodrop spectrophotometer (LabGenius SQ2802DS, Thermofisher Scientific). Integrity was confirmed by measuring DNA concentration; samples were acceptable if C-DNA was at least 10 µg/dL. Purity was evaluated by the absorbance ratio (A260/A280), with acceptable values between 1.0 and 2.0. Only samples meeting both standards were used in further molecular analyses.

### 2.5. DNA Amplification

After confirming DNA integrity and purity, the samples were subjected to PCR amplification using an Eppendorf Mastercycler Pro thermal cycler Thermofisher Scientific). The amplification protocol consisted of an initial denaturation at 95 °C for 5 min, followed by 30 cycles of denaturation at 95 °C for 15 s, annealing at 61.5 °C for 15 s, and extension at 72 °C for 30 s. A final extension step at 72 °C for 5 min was included to ensure complete amplicon synthesis.

The reaction mixture (final volume: 20 µL) was prepared using 12.5 µL of GoTaq^®^ Probe qPCR Master Mix (Promega, Madison, WI, USA), Taq-Polymerase, Taq-&LOAD 5×C buffer (MP Biomedicals, Sata Ana, CA, USA), and 5.5 µL of nuclease-free water. For each reaction, 1 µL of template DNA, 1 µL of forward primer, and 1 µL of reverse primer were added. The primers used to amplify 16S rRNA regions specific to Firmicutes and Bacteroidetes, as previously described by Grigoreva [13], are listed in Table 1.

Following amplification, PCR products were stored at −20 °C until electrophoretic verification and subsequent quantification of the Firmicutes/Bacteroidetes ratio.

### 2.6. Determination of the Firmicutes/Bacteroidetes Ratio

The relative abundance of Firmicutes and Bacteroidetes was estimated by quantifying the PCR-amplified products through agarose gel electrophoresis. PCR products were loaded onto a 1% (*w*/*v*) agarose gel prepared with Tris–acetate–EDTA (TAE) buffer and stained with a nucleic acid intercalating dye. Electrophoresis was performed at 100 V for 30 min using a standard horizontal gel system, allowing separation of amplicons by molecular size.

After completing the run, the gels were transferred to a Gel Doc™ EZ Imager (Bio-Rad, Hercules, CA, USA) for digital visualization. Band intensity was measured using Image Lab software (version 3.0) after automated background subtraction and normalization. The concentration of each amplicon is expressed in relative optical density units.

The Firmicutes/Bacteroidetes (F/B) ratio was calculated individually for each participant by dividing the band intensity of Firmicutes amplicons by that of Bacteroidetes. Subsequently, group-level values were obtained by calculating the mean of individual F/B ratios, rather than deriving ratios from averaged phylum-level values. This approach minimizes potential bias associated with aggregating independent measurements.

### 2.7. Data Processing

Descriptive statistics were obtained through univariate analysis, including frequencies, minimum and maximum values, means, and standard deviations (SDs), using SPSS version 25. For inferential analyses, the distribution of all variables, including the F/B ratio, body fat percentage, glucose, triglycerides, total cholesterol, and HDL-C, was first assessed using the Kolmogorov–Smirnov test.

Linear regression models were also built to estimate the coefficient of determination and examine the predictive relationships between the F/B ratio and metabolic indicators. Variables that did not meet normality assumptions were examined with Spearman’s rank correlation. These analyses evaluated the strength and direction of the relationships between the F/B ratio and each biochemical measurement and body fat percentage.

All statistical procedures were performed using Jamovi version 2.3.21.0, with a significance level set at *p* < 0.05.

## 3. Results

### 3.1. Anthropometric Data

Table 2 shows the demographic and anthropometric characteristics of the study population. The age ranged from 18 to 25 years, with 23 years being the most common (22.9%) and 18 years being the least common (1.4%). Among the 70 participants, females made up slightly more than half (55.7%) compared to males (44.3%).

According to BMI classification, most individuals fell within the normal weight range (61.4%), followed by overweight (25.7%) and obese (12.9%). However, when categorized by body fat percentage, the distribution shifted notably: 34.3% of participants were considered normal weight, 25.7% were overweight, and 40% were obese. This difference between BMI and body fat percentage classifications highlights the limitations of BMI in accurately reflecting adiposity, especially in young populations, and underscores the importance of including direct body composition assessments in metabolic and microbiota research.

### 3.2. Results of the Biochemical Parameters

The biochemical parameters of the study population are shown in Table 3. The mean fasting glucose was 83.99 ± 7.78 mg/dL, remaining within clinically normal ranges for all participants. Total cholesterol levels also fell within expected values, with an average of 172.71 ± 29.7 mg/dL. Triglycerides showed greater variability, with a mean of 116.35 ± 83.7 mg/dL, indicating that some participants had elevated levels despite the overall mean staying close to normal reference limits.

HDL-C levels varied by sex, as expected: women had a mean concentration of 57.74 ± 11.56 mg/dL, while men had a mean concentration of 51.38 ± 7.88 mg/dL. Both values are within desirable ranges, although the wide variation observed—especially in triglycerides—indicates diverse metabolic profiles within the cohort. These findings provide an important metabolic baseline for understanding the relationships among biochemical markers, body composition, and gut microbiota composition.

### 3.3. Firmicutes/Bacteroidetes Ratio

Table 4 summarizes the volumes of the Firmicutes and Bacteroidetes amplification bands, measured in milli-absorbance units (mAUs), across groups classified by BMI and fat mass percentage. When categorized by BMI, the Firmicutes band volume showed similar values in the normal weight group (46.9 ± 21.2 mAU) and the overweight group (46.3 ± 13.6 mAU), reaching its lowest in the obesity group (37.2 ± 16.9 mAU). Conversely, the highest value in the Bacteroidetes band volume was exhibited in the obese group (62.7 ± 16.9 mAU), while in the overweight and normal weight groups, the values were considered close (53 ± 21.2 mAU and 53.6 ± 13.6 mAU, respectively). These variations contributed to an increased Firmicutes/Bacteroidetes (F/B) ratio in both overweight (1.3) and obese (1.3) participants compared with the normal-weight group (0.84).

A similar trend was observed when participants were grouped by percentage of fat mass. Firmicutes volume increased significantly from the fat group (82.15 ± 7.36 mAU) to overweight levels of adiposity (191.54 ± 21.08 mAU), while Bacteroidetes values remained more stable across categories. As a result, the F/B ratio rose considerably in the overweight-fat group (1.14) compared to the lower ratio observed in individuals with average fat mass (0.46). Although the obesity-fat-mass group showed higher absolute volumes of both phyla, the resulting ratio (0.89) remained intermediate.

Overall, these findings show that increases in adiposity, whether measured by BMI or body fat percentage, are linked to a relative increase in Firmicutes abundance, as reflected in higher F/B ratios in groups with more body fat.

After conducting the nonparametric Kruskal–Wallis test to compare means, no significant differences were found in any of the test results. The results demonstrated that the mean Firmicutes/Bacteroidetes ratio increased proportionally with both BMI and body fat percentage.

### 3.4. Relationships Between the F/B Ratio and Biochemical Parameters

**Figure 1:**
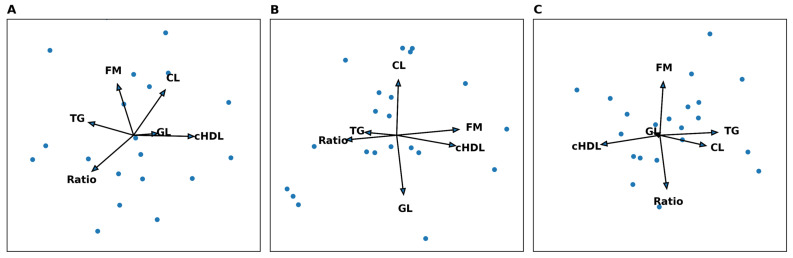
Linear regression analysis of metabolic variables and the Firmicutes/Bacteroidetes ratio in normal weight (**A**), overweight (**B**), and obese participants (**C**). TG: Triglycerides; CL: Cholesterol; GL: Glucose; FM: %fat mass.

Figure 1 illustrates the linear regression analysis of metabolic variables and the Firmicutes/Bacteroidetes ratio. In the normal weight group (A), the vectors for total cholesterol, glucose, and c-HDL point in different directions, indicating weak or no correlations among these parameters. The triglyceride vector points opposite to c-HDL, consistent with the expected inverse relationship between these lipid markers. The Firmicutes/Bacteroidetes (F/B) ratio vector shows an independent direction from the metabolic indicators, suggesting no strong link between gut microbial composition and biochemical variables in this group. The wide distribution of points also reflects high individual variability.

In the overweight group (B), a clearer clustering pattern emerged, with total cholesterol and triglycerides pointing in similar directions, indicating a positive relationship. c-HDL showed an opposite orientation to triglycerides, as expected in dyslipidemic trends. Glucose exhibited little alignment with lipid markers. The F/B ratio continued to be largely independent of the metabolic variables, with vectors indicating minimal relation.

In the obesity group (C), the link between cholesterol and triglycerides became clearer, matching traditional dyslipidemic patterns. The c-HDL vector stayed separated from triglycerides, showing an inverse relationship. Both glucose and the F/B ratio showed directions mostly perpendicular to the lipid markers, emphasizing the lack of a consistent link between microbiota makeup and biochemical measures in people with obesity. There was still much variation among participants, indicating high metabolic diversity.

The linear regression analysis confirmed that the relationships between the F/B ratio, metabolic indicators, and body fat percentage were generally weak, with all models producing nonsignificant *p* values (*p* > 0.05). Similarly, correlations based on BMI-classified groups were also nonsignificant. However, a slight increase in correlation strength was noted between body fat percentage and the F/B ratio in the overweight (r = 0.38) and obesity groups (r = 0.33). For other variables, correlation coefficients ranged from 0.000 to 0.251, indicating very weak associations.

Performing Spearman analysis between body composition, lipid profile, and gut microbiota variables revealed some significant correlations (Table 5). Body fat percentage showed moderate positive correlations with triglycerides (ρ = 0.272, *p* = 0.023) and total cholesterol (ρ = 0.304, *p* = 0.011), suggesting a direct relationship between adiposity and lipid metabolism alterations.

Triglycerides were positively correlated with total cholesterol (ρ = 0.449, *p* < 0.001) and negatively correlated with HDL cholesterol (ρ = −0.536, *p* < 0.001), indicating an atherogenic lipid profile characterized by dyslipidemia. The inverse association with HDL-c supports its protective role against metabolic disturbances.

Regarding gut microbiota, HDL-c showed a negative correlation with the Firmicutes/Bacteroidetes (F/B) ratio (ρ = −0.243, *p* = 0.043), suggesting that an increased F/B ratio may be associated with a less favorable lipid profile. Additionally, internal relationships between bacterial phyla were consistent: Firmicutes were positively correlated with Bacteroidetes (ρ = 0.649, *p* < 0.001) and with the F/B ratio (ρ = 0.449, *p* < 0.001), while Bacteroidetes were negatively correlated with this ratio (ρ = −0.314, *p* = 0.008), as expected given that this index reflects their relative abundance.

Although overall correlations remained weak, glucose and body fat percentage showed slightly higher coefficients in the overweight and obese BMI groups. Conversely, triglycerides, total cholesterol, and c-HDL demonstrated comparatively higher correlations within groups classified by body fat percentage, indicating that biochemical–microbiota relationships might be more evident when adiposity is used as the main stratification variable instead of BMI. In addition to subgroup analyses, overall trends across the dataset were evaluated and showed similarly weak and non-significant associations.

## 4. Discussion

The present results showed that as body weight and body fat percentage increased, the relative abundance of both Firmicutes and Bacteroidetes also increased, a pattern reflected in gradual changes in the Firmicutes/Bacteroidetes (F/B) ratio. Although several studies have associated dysbiosis with higher Firmicutes levels, the direction and magnitude of these changes remain inconsistent. For instance, Duan et al. [17] reported an increase in Bacteroidetes and a decrease in Firmicutes in obese individuals, emphasizing that differences in the gut microbiota may be more pronounced at the species level than across entire phyla, as evidenced by the reduction in 12 Firmicutes species in their obese group.

It is important to note that quantifying individual phyla based on PCR band intensity can be affected by technical variability, including differences in DNA extraction efficiency, PCR amplification success, and the presence of inhibitors. Therefore, absolute or relative comparisons of Firmicutes and Bacteroidetes alone should be approached with caution. In this context, the Firmicutes/Bacteroidetes (F/B) ratio might serve as a more consistent metric, since both values are obtained from the same sample and are subject to similar technical variations. As a result, the results concerning individual phyla in this study are presented descriptively and should not be overinterpreted in terms of biological significance.

Similarly, studies by Qadir et al. [18] and Palmas et al. [19] reported increased Firmicutes and decreased Bacteroidetes in obese individuals; however, Qadir et al. [18] observed these differences without statistical significance (*p* = 0.054). Palmas et al. [19] further suggested that weight gain may selectively affect species within each phylum, which could partially explain the variability and inconsistency across studies [18]. These findings underscore the limitations of the F/B ratio as a standalone marker, as it may mask biologically relevant shifts at lower taxonomic levels.

Within the same population, the relative abundance of Firmicutes and Bacteroidetes varies and is influenced by lifestyle factors, including diet, physical activity, food additives, environmental contaminants, and antibiotic exposure [20]. In the present study, these influences were reduced by selecting a relatively homogeneous sample, thereby minimizing variability associated with age and lifestyle [21]. Nevertheless, evidence from the Mexican population indicates that microbial associations may differ by metabolic phenotype, with Firmicutes and Bacteroidetes linked to obesity in some subgroups and to triglyceride levels in others. Specifically, *Verrucomicrobia* has been negatively associated with obesity and positively correlated with genera such as *Bacteroides*, *Alistipes*, and *Clostridium*, whereas in obese individuals, genera including *Fusicatenibacter*, *Romboutsia*, *Ruminococcaceae*, *Ruminiclostridium*, *Blautia*, *Clostridium*, *Anaerostipes*, and *Intestinibacter* have been associated with both obesity and plasma insulin levels [22]. This heterogeneity further limits the interpretability of the F/B ratio in isolation.

The F/B ratio values observed in this study are comparable to those reported in populations stratified by BMI and age [23]. Although these results differ from those reported by Mariat et al. [24], it is well recognized that variations in the F/B ratio are influenced by age, body weight, environment, genetic background, and methodological approaches [24,25]. In this context, the alignment of our findings with those reported by Vaiserman et al. [25] supports the internal consistency of the association analyses and highlights population-specific differences that limit the generalizability of the F/B ratio.

The relationship between gut microbiota composition and glucose metabolism is bidirectional, with glucose availability influencing bacterial colonization as a key metabolic substrate [26]. Comparative studies of normal-weight and obese individuals have reported reductions in Firmicutes, particularly in species such as *Faecalibacterium prausnitzii* [27]. However, Qin et al. [22] demonstrated that microbial alterations associated with impaired glucose tolerance are more evident at the species level, including reductions in butyrate-producing bacteria such as *Akkermansia muciniphila* and *Faecalibacterium prausnitzii*, which decline as glucose metabolism worsens [22,28]. The inverse association of these beneficial microbes with obesity and diabetes highlights their metabolic relevance, which is not adequately captured by phylum-level metrics such as the F/B ratio [29].

The gut microbiota composition also influences lipid metabolism. Obesity-related dysbiosis is often described as a decrease in Bacteroidetes and an increase in Firmicutes; however, human studies report considerable variability in these patterns [30]. Associations have been identified between lipid profiles and specific bacterial taxa, including *Akkermansia*, *Christensenellaceae*, and *Tenericutes*. While obesity has been linked to higher triglycerides, HDL-C, and BMI, taxa such as Eggerthella, Pasteurellaceae, and Butyricimonas have been associated with these parameters. In contrast, total cholesterol and LDL-C appear less consistently related to dysbiosis, suggesting differential microbial influences on lipid fractions [31].

In the present study, linear regression analysis of the F/B ratio and lipid profile indicators (triglycerides, total cholesterol, and HDL-C) showed weak, statistically non-significant associations across groups classified by body fat percentage and BMI. Although some improvement in the strength of relationships was observed across classification methods, these inconsistencies underscore the limited sensitivity of the F/B ratio in detecting metabolic alterations in young adults.

The Spearman analysis suggested an interaction among body composition, lipid metabolism, and the gut microbiota, in which increased adiposity and a higher F/B ratio may contribute to an unfavorable metabolic profile. However, the strength of some correlations is moderate, indicating that these relationships should be interpreted within a multifactorial context.

Given that approximately 90% of the gut microbiota consists of Firmicutes and Bacteroidetes [14], the functional diversity within these phyla—particularly within Firmicutes—has led to the proposal of the F/B ratio as a biomarker of obesity-related dysbiosis [13]. However, systematic reviews and meta-analyses have reported increased Firmicutes without consistent reductions in Bacteroidetes, along with substantial shifts at the genus level, including decreased *Bifidobacterium* and *Eggerthella* and increased abundance of several other genera in obese individuals [32,33]. Despite a general reduction in microbial diversity in obesity, marked heterogeneity across studies limits the establishment of a robust, reproducible F/B-based association. Methodological improvements in DNA extraction, amplification, and sequencing strategies have been recommended to enhance taxonomic resolution and reproducibility [34,35].

Comparisons across studies are further complicated by inconsistent population characterization and inadequate control of confounding factors [21]. Many studies lack detailed information on antibiotic exposure, probiotic or prebiotic intake, diet composition, and physical activity levels, all of which can profoundly influence gut microbiota composition [32]. It remains unclear whether the microbiota alterations observed in obesity are driven primarily by dietary patterns or by obesity itself. Additional factors, including nondigestible carbohydrates, protein sources, unabsorbed minerals, phenolic compounds, dietary lipids, food additives, and environmental pollutants, also modulate microbial communities and metabolite production [33]. Careful consideration of these variables is essential to avoid biased interpretations when comparing populations with distinct lifestyles [21]. A key limitation of this study is the absence of dietary intake data. Since diet significantly affects gut microbiota composition and function, not accounting for it may have contributed to the variability seen in the Firmicutes/Bacteroidetes ratio among individuals. Even though the study group was fairly consistent in age and academic setting, unmeasured dietary habits could have confounded microbiota profiles and their links to metabolic parameters.

Although the mean F/B ratio increased across groups classified by body fat percentage and BMI, the weak correlations observed in this study support the conclusion that the F/B ratio alone is an insufficient indicator of metabolic status in young adults. These findings align with evidence from systematic reviews and meta-analyses that emphasize the importance of methodological factors, including DNA source, primer selection, and bioinformatics pipelines. Future research should prioritize integrative microbiota-based approaches that incorporate species-level resolution, functional analyses, and detailed lifestyle characterization to better elucidate relationships between gut microbiota composition and metabolic health [21].

This study has several limitations. The sample size calculation was based on population estimates rather than comparisons between groups, which may limit the statistical power to detect differences among study groups. Additionally, the relatively small sample size (*n* = 70) may have limited the ability to detect subtle associations. The restricted age range (18–25 years) also limits the generalizability of the findings to other populations. Furthermore, although some sex-related differences were observed (e.g., HDL-C levels), a detailed stratified analysis by sex was not conducted, which might have limited the detection of sex-specific associations. Moreover, key confounding factors, such as diet and physical activity, were not controlled and could have influenced gut microbiota composition. Biochemical parameters were measured at only one time point, which limits understanding of changes over time. Body fat percentage was assessed using bioelectrical impedance instead of gold-standard methods like dual-energy X-ray absorptiometry. Lastly, microbiota analysis was limited to phylum-level quantification, which does not account for functional or species-level differences.

## 5. Conclusions

Although the Firmicutes/Bacteroidetes (F/B) ratio increased gradually across groups classified by body fat percentage and nutritional status, its association with metabolic indicators and adiposity was weak and not statistically significant. These findings indicate that the F/B ratio alone does not adequately reflect metabolic status in young Mexican adults. While this study contributes to characterizing gut microbiota distribution in this population, the observed patterns suggest that obesity-associated dysbiosis cannot be reliably inferred from phylum-level ratios.

Marked interindividual variability in the abundance of Firmicutes and Bacteroidetes is strongly influenced by dietary habits, physical activity, food additives, environmental contaminants, and antibiotic exposure, all of which modulate gut microbiota composition. This variability likely underlies inconsistencies across studies and limits the ability to establish direct links between the F/B ratio and specific metabolic outcomes. Although the gut microbiota may contribute to obesity-related mechanisms, current evidence remains insufficient to support the F/B ratio as a standalone biomarker.

Future research should prioritize comprehensive characterization of subjects and the identification of lifestyle, environmental, and methodological factors that shape microbiota composition to improve the interpretation of microbiome–metabolic relationships. Given the complexity of obesity–gut microbiota interactions, reliance on a single taxonomic marker is overly simplistic. Rather than supporting the use of the F/B ratio as a biomarker, our findings highlight its limitations and reinforce the need for integrative, high-resolution approaches that incorporate species-level data, functional profiling, and host metabolic parameters.

## Figures and Tables

**Table 1:** Primers used for the amplification of Firmicutes and Bacteroidetes 16S rRNA genes.

Target Group	Primer	Sequence (5′–3′)
Bacteroidetes	Forward (798CFBf)	AAACTCAAAKGAATTGACGG
	Reverse (cfb967R)	GGTAAGGTTCCTCGCGCTAT
Firmicutes	Forward (928F-firm)	TGAAACTYAAGGAATTGACG
	Reverse (1040fIRMr)	ACCATGCACCACCCTGTC

**Table 2:** Description of the study population included within the group of young Mexican university students.

Variable	Frequency		Percentage (%)
Age	18	1	1.4
	19	2	2.9
	20	4	5.7
	21	12	17.1
	22	15	21.4
	23	16	22.9
	24	11	15.7
	25	9	12.9
Gender	Female	39	55.7
	Male	31	44.3
Classification by BMI	Regular weight	43	61.4
	Overweight	18	25.7
	Obesity	9	12.9
Classification by percentage of fat	Regular weight	24	34.3
	Overweight	18	25.7
	Obesity	28	40.0

**Table 3:** Descriptive analysis of metabolic indicators of young Mexican university students.

Variable	GLU (mg/dL)	TG (mg/dL)	TC (mg/dL)	c-HDL^M^ (mg/dL)	c-HDL^H^ (mg/dL)
Median	83.1 ± 5.78	116.35 ± 83.77	172.71 ± 29.73	57.74 ± 11.56	51.38 ± 7.88
Range	70–95	31–518	105–247	35–79	38–70

where GLU—glucose, TG—triglycerides, TC—total cholesterol, c-HDL^M^—cholesterol in women, and c-HDL^H^—cholesterol in men.

**Table 4:** Volume (mAU) of the Firmicutes and Bacteroidetes amplification band by study groups classified by BMI and % of fat mass (FM).

BMI			
**Group**	**Normal Weight**	**Overweight**	**Obesity**
Firmicutes	46.9 ± 21.2	46.3 ± 13.6	37.2 ± 16.9
Bacteroidetes	53 ± 21.2	53.6 ± 13.6	62.7 ± 16.9
Ratio F/B	1.3	0.98	0.80
**% Fat Mass (FM)**			
Firmicutes	39.7 ± 17.3	46.3 ± 22.3	49.9 ± 17.6
Bacteroidetes	60.2 ± 17.3	53.6 ± 22.3	50 ± 17.6
Ratio F/B	0.84	1.3	1.3

The F/B ratio values correspond to the mean of individual ratios calculated for each participant and not to ratios derived from averaged Firmicutes and Bacteroidetes values.

**Table 5:** Significant correlations between clinical, metabolic variables, and intestinal microbiota after Spearman analysis (*n* = 70).

Variable 1	Variable 2	ρ (Rho)	*p*-Value
Body fat percentage	Triglycerides	0.272	0.023
Body fat percentage	Total cholesterol	0.304	0.011
Triglycerides	Total cholesterol	0.449	0.000
Triglycerides	HDL-c	−0.536	0.000
HDL-c	F/B ratio	−0.243	0.043
Firmicutes	Bacteroidetes	0.649	0.000
Firmicutes	F/B ratio	0.449	0.000
Bacteroidetes	F/B ratio	−0.314	0.008

## Data Availability

The original contributions presented in this study are included in the article. Further inquiries can be directed to the corresponding author.

## Abbreviations

The following abbreviations are used in this manuscript:

BMI	Body Mass Index
F/B	Firmicutes/Bacteroidetes ratio
C-DNA	DNA concentration
CHO-POD	Cholesterol oxidase/peroxidase
C-PRO	Concentration ratio
GPO-POD	Glycerol phosphate oxidase–peroxidase
HDL-C	High-density lipoprotein cholesterol
NADPH	Nicotinamide adenine dinucleotide phosphate
NADP^+^	Nicotinamide adenine dinucleotide phosphate oxidized
SD	Standard deviation
TAE	Tris-acetate-EDTA
